# Decolonization of *Staphylococcus aureus* in Healthcare: A Dermatology Perspective

**DOI:** 10.1155/2018/2382050

**Published:** 2018-12-24

**Authors:** Drew Kuraitis, Laura Williams

**Affiliations:** Department of Dermatology, Tulane University, New Orleans, LA, USA

## Abstract

The bacterium *Staphylococcus aureus* is responsible for significant morbidity, mortality, and financial burden in healthcare. It easily colonizes susceptible patients and can cause recurrent infections, especially in populations at risk. In addition to treating sequelae of infections, there is a growing body of literature aimed at decolonizing susceptible patients in order to prevent infection and also to prevent spread. Such strategies are widely employed in surgical, intensive care, and hospitalist fields. *Staphylococcus aureus* involvement has been implicated in the pathogenesis and persistence of many dermatologic diseases that are treated in the outpatient setting. This review serves to summarize current evidence for the management of *Staphylococcus aureus* colonized patients, as well as the evidence available for decolonization. We further characterize the role that colonization may play in atopic dermatitis, recurrent infections, hand eczema, cutaneous T-cell lymphoma, and also in surgical infections after Mohs surgery.

## 1. The Role of *Staphylococcus aureus* in Healthcare

*Staphylococcus aureus* is considered normal flora of healthy mammals. In humans, the nose and pharynx are typical sites of colonization, with the nasal vestibule as the main reservoir [1, 2]. Overall carrier rates in healthy humans range from 20 to 50%. Studies have shown that colonizing strains of *S. aureus* are the same as strains isolated from local skin and soft tissue infections (SSTIs) [3]. After leaving its colonization site, *S. aureus* can infect any body part or organ system. It is the most common pathogen involved in SSTIs, is now the most common invasive pathogen in the United States [4], and is also responsible for 2% of hospital admissions [5]. Risk factors for colonization and/or *S. aureus* infections include hospital or nursing home exposure, immunodeficiency, chronic illness, poor hygiene, working with hogs, and being a household cohabitant of a colonized individual [6]. Due to its prevalence, associated morbidity, and ease of transmission, it is therefore beneficial from individual, epidemiological, and financial perspectives to intervene. A growing body of literature is focused on attempting to decolonize individuals in order to achieve this. If we can rid a carrier of colonizing *Staphylococcus*, we can improve morbidity, spread of disease, and the associated financial burden.

One of the major challenges when treating *S. aureus* is antibiotic resistance. Shortly after their clinical debut, methicillin and penicillin became ineffective against methicillin-resistant *Staphylococcus aureus* (MRSA) [7]. MRSA now compromises at least 60% of *Staphylococcus* isolates from intensive care units. It is suspected that current decolonization efforts may not be as effective when dealing specifically with MRSA [8]. On admission to the hospital, patients after often subjected to a nasal swab to identify MRSA carriers, but this procedure may miss up to 50% of carriers to colonization of extranasal sites [9]. Many agents used in decolonization are presumed immune to bacterial resistance, offering some advantages when attempting to decolonize MRSA. Literature is rich in meta-analyses and systematic reviews for managing *Staphylococcus* colonization in patients undergoing dialysis [10], orthopedic surgery [11, 12], cardiac surgery [13, 14], plastic surgery [15], and general surgery [16, 17]. To date, there is not a published review regarding this topic in dermatology. This review is intended to summarize the role that *S. aureus* carriage may play in common dermatologic conditions, as well as what evidence is available for dermatologists when recommending decolonization protocols.

## 2. *Staphylococcus* Colonization in Dermatology

*S. aureus* colonization has been implicated in the pathogenesis or persistence of many skin diseases, for which physicians will often recommend decolonization regimens. Unfortunately, randomized controlled trials (RCTs) investigating the role of decolonization in these diseases are lacking in literature, and there is no consensus as to the ideal decolonization protocol. We have summarized key points on what has been shown so far in literature with respect to common skin conditions that have been linked to staphylococcal colonization.

### 2.1. Atopic Dermatitis

Atopic dermatitis (AD) is a relatively common skin condition, with recent prevalence estimates of 15–20% in children and 2–10% in adults [18]. Strong associations exist between recurrent skin infections, disease severity, and *S. aureus* colonization [19, 20]. Individuals with AD have a greater concentration of *S. aureus* colonization of their skin [21]. Colonization of AD skin lesions can not only lead to skin infections in these patients but may also exacerbate the disease, further leading to infection and thereby contributing to chronic disease and frequent flares [22, 23]. Studies have shown that at least 80% of patients with AD are colonized by *S. aureus*, with up to 30% of the colonizing strains being MRSA [24, 25]. A recent systematic review pooled 95 studies and concluded that 70% of lesional skin is colonized, compared to 39% of nonlesional skin, and that 62% of patients with AD have nasal colonization. Furthermore, they also correlated prevalence of colonization with disease severity [26]. A prospective study that followed 605 pregnant women sought to determine whether or not *S. aureus* colonization can predict development of AD. They found that, in infants who developed AD, there is a marked increase in *S. aureus* colonization at age 3 months and also a greater prevalence 2 months prior to the diagnosis of AD, suggesting that *S. aureus* may have a role in the development and onset of AD in addition to its known role of exacerbating AD [27].

Although the pathogenesis of AD is multifactorial, patients overall remain at greater risk of skin and soft tissue infections due to skin changes that render them more susceptible. Such changes include the decreased level of barrier lipids [28, 29], increased local serum proteases [30], and reduced antimicrobial peptides, such as beta-defensin [31, 32]. Another important molecule which appears to be involved in *S. aureus*-AD interplay is filaggrin (FLG). FLG binds keratin and is important for barrier protection. It exerts its effect by promoting formation of the stratum corneum, reducing water loss, and regulating pH [33]. Some patients with AD have mutations in FLG and ultimately experience more severe disease. Individuals with FLG mutations have been associated with *S. aureus* colonization and disease severity, highlighting the relationship between genetics and the skin microbiome [34, 35]. *S. aureus* is also able to modulate the host environment, largely through superantigens, which disrupt the skin barrier by increasing proinflammatory cytokine production by keratinocytes [36]. The AD host environment appears to promote bacterial colonization, and in-turn, the bacteria aggravate the disease, propagating a pathogenic cycle. Therefore, breaking this cycle using decolonization methods may serve to lessen the severity of AD.

Sodium hypochlorite, or bleach, has been used in medicine as an antiseptic for centuries [37]. Bleach has broad-spectrum antimicrobial activity, including MRSA coverage, and does not carry the risk of antimicrobial resistance [37, 38]. Dilute bleach baths are a staple recommendation by dermatologists for the treatment of atopic dermatitis. It is thought that bleach baths can decrease colonization and recurrent skin infections in those with AD, thereby reducing disease severity and improving quality of life [39]. Bleach is also attractive due to its low cost, ease of access, and tolerability [37]. Despite initial studies showing promising results, a modified Cochrane review assessed RCTs that investigated whether or not AD can clinically improve using antistaphylococcal treatments and concluded that interventions such as bleach baths and topical antiseptics provide no clinical benefit for individuals who do not have evidence of current infection [40]. Of the 26 RCTs (*n*=1229) that were analyzed, many of them had study-design limitations relating to lack of randomization method description, small sample sizes, or not describing baseline AD severity. Regardless, their systematic review does not dispute that antimicrobial interventions are successful in reducing the bacterial burden in AD and that this burden indeed plays a role in pathogenesis.

One RCT recruited 31 patients with AD who had clinical signs of bacterial secondary infection. They were treated with bleach baths twice per week and intranasal mupirocin twice per day for 5 days each month. Controls received vehicle alone. After 3 months, individuals in the treatment arm had a significant reduction in the amount of body surface area affected and reduced severity of their AD [41]. A similar RCT that focused on 200 patients with skin and soft tissue infections compared the decolonization ability of intranasal mupirocin alone for 5 days and intranasal mupirocin plus daily dilute bleach baths for 5 days. After 4 months, the study reported that combined treatment eradicated colonizing *S. aureus* in 71% of patients, compared to 48% in the mupirocin-alone group [42]. This study also included a control “education only” group. All control and treatment groups received education on hygiene, which stressed discarding lotions in jars, replacing lotions with pump or pour bottles, refraining from sharing personal items such as razors or towels, and washing bed linens at least once weekly and towels after each use. 38% of individuals in the “education only” group were reportedly cleared of colonizing bacteria. Although it appears that a combination of intranasal mupirocin and bleach baths may be effective at reducing bacterial colonization, proper sanitation alone may play an important role in decolonization. Given the vast fund of knowledge regarding the microbiome's interactions with AD, it would be helpful if future studies correlate antimicrobial treatments and disease severity with the degree of colonization.

### 2.2. Recurrent Skin and Soft Tissue Infections

The incidence of *Staphylococcus*-associated SSTIs continues to rise [43]. SSTIs include abscesses, furunculosis, and cellulitis, and this group is now included in the 10 most common reasons for hospital admission [44]. Decolonization strategies are often recommended as a way to prevent recurrent SSTI. Common regimens again include bleach baths, intranasal mupirocin, and chlorhexidine body washes. A survey of healthcare providers revealed that the majority (53%) of providers treated recurrent SSTIs with the same antibiotic that was previously used for the same duration of time; providers of adult patients favored trimethoprim-sulfamethoxazole (TMP-SMX) and those of children favored clindamycin. Regarding decolonization recommendations, the top 3 antimicrobial therapies included mupirocin (88%), antiseptic body wash (79%), and bleach baths (34%) [45].

The previously mentioned study that found that intranasal mupirocin with bleach baths was superior to intranasal mupirocin or education alone for reducing colonization was focused on individuals with SSTIs [42]. One of the limitations to this study was that across all treatment arms, 20% of patients reported a recurrent SSTI one month after treatment. Another limitation was the lack of household decolonization. It is plausible that the benefits observed could have been sustained if the decolonization treatment was completed on a regular basis, or if household members could participate in the process as well. Risk factors for *Staphylococcus* colonization include household contact with someone who had a recent SSTI [46, 47]. An RCT of 183 individuals with a recent *S. aureus* abscess sought to compare the effectiveness of individual vs household decolonization on *Staphylococcus* carriage and recurrent SSTI [48]. After twice daily intranasal mupirocin and daily chlorhexidine washes for 5 days, the authors found no difference in the rate of colonization, but they did note that at 12 months, there were less reports of SSTIs in those undergoing household decolonization (52% vs 72%), suggesting a long-term benefit of household decolonization.

One study attempted to eradicate *Staphylococcus* colonization in patients with recurrent SSTIs by treating with a prolonged course of chlorhexidine wash twice per day for 60 days and 30 days of one oral antibiotic, as determined by the clinician. Although they reported impressive clearance rates of up to 90% at 4 months follow-up, this study lacked a control arm and also was unable to standardize the oral antibiotic regimen given to subjects [49]. The authors also acknowledge that their treatment course is longer than other regimens that have been studied, but that this may be justified in the treatment of recurrent staphylococcal SSTIs. Decolonization with the intent to reduce SSTIs has been extensively studied in military populations, as new recruits are at a high risk of developing SSTI [50]. SSTI is the leading cause of hospital admission in the first two years of a new recruit's military career. One study followed over 33,000 recruits to a 13-week training course and demonstrated that showering 5 to 6 times with a chlorhexidine wash was able to reduce SSTI incidence [51]. This was in contrast with another study of over 30,000 recruits using chlorhexidine wash weekly, which did not demonstrate any benefit [52]. The latter study did not have recruits wash with chlorhexidine on arrival, which may account for the study differences. A secondary analysis of the latter study correlated reduced MRSA colonization of the nares with those who frequently used the chlorhexidine wash, despite the initial study not demonstrating a reduction in SSTI [53].

The presentation of an individual with a skin or soft tissue abscess is a common situation encountered in emergency rooms. The Infectious Disease Society of America's guidelines for management of an uncomplicated abscess are incision and drainage (I&D) alone. Although the use of antibiotics in addition to I&D is controversial and reviewed elsewhere [54], two RCTs looking at abscess treatment with I&D with or without the antibiotic TMP-SMX did not identify differences in percentage of treatment failures or recurrent infections, demonstrating noninferiority. However, these studies followed patients longitudinally and suggest that antibiotic therapy after I&D prevented recurrent abscess formation at a new, distinct site. The pediatric study surveyed patients 10 days after treatment and found that 12.9% of those receiving antibiotics developed a new abscess, compared to 26.4% in the placebo group [55]. The other study followed adult patients to 30 days after I&D and found that 9% of those receiving TMP-SMX reported new lesions compared to 28% of those receiving placebo [56]. More evidence for the role of oral antibiotics in preventing recurrent SSTIs comes from a study of 357 *S. aureus*-colonized children requiring I&D for an abscess [57]. 331 of subjects received adjuvant clindamycin or TMP-SMX and 26 received no antibiotics. The mean follow-up appointment was 38 days later, when swabs were taken to determine if patients were still colonized and colonization rates were higher in those not treated with antibiotics. At 1 year follow-up, 57% of those who were colonized with *S. aureus* at the “38-day” follow-up reported a recurrent SSTI compared to 30% of those who were not colonized at follow-up. This study is of importance because it demonstrated that the administration of systemic antibiotics at the time of I&D may reduce colonization and is protective against recurrent infection. In the current era of antibiotic resistance, it seems unlikely that oral antibiotics will become standard of care for decolonization protocols. It is, however, important to note that oral antibiotics can reduce recurrent infections when considering risks and benefits of adjunct antibiotic therapy for SSTIs and abscesses.

### 2.3. Hand Eczema

Hand eczema is a chronic dermatitis characterized by dry, painful, cracked skin on the hands with or without blisters and weeping. It tends to be secondary to an irritant, such as gloves, vegetables, disinfectants, or other ingredients in personal care products. It is more common in people who frequently wash their hands, such as healthcare workers and chefs, but it is also increased in individuals with a history of AD [58, 59]. There have been two studies looking at the role *S. aureus* colonization may play in hand eczema. The first study demonstrated that patients with hand eczema were more likely to have *Staphylococcus* colonization on their hands and that this was associated with severity of disease [60]. The other study used a “glove juice” method where patients with severe hand dermatitis placed their hands in a loose-fitting glove and saline was used to rinse the entire hand, with analysis of the wash. This method helps to reduce sampling bias since the entire surface of the hand can be tested in this way. Not surprisingly, they found a positive association with severity of hand eczema and density of colonizing *S. aureus* in addition to increased baseline levels of colonization in those affected compared to healthy controls [61]. It is also notable to point out that neither of these studies showed a difference in nasal colonization when comparing patients with hand eczema and those without. A 2012 study found Gram-positive cocci contamination in 90% of topical medications and creams used by hand eczema patients, with 30% of these organisms being *S. aureus* [62]. Other studies have found similar results with cosmetics and creams in jar format, further highlighting the need for proper education regarding hygiene precautions [63]. Taken together, these studies suggest a role of *S. aureus* in the pathogenesis and/or persistence of hand eczema and that there may be clinically relevant exogenous sources of microbes in addition to nasal colonization, which is not significantly increased in patients with hand eczema. There is a lack of studies investigating the potential of decolonization in hand eczema management.

### 2.4. Cutaneous T-Cell Lymphoma

Cutaneous T-cell lymphoma (CTCL) is a clonal T-cell proliferation where malignant cells localize to the skin [64]. These cells can be characterized by their T-cell receptor variable region beta-chain (V*β*) expressions [65]. T-cells expressing particular V*β* variations can proliferate in response to *Staphylococcus* exotoxins and superantigens *in vitro* [66]. Sezary cells isolated from patients with CTCL have also been observed to proliferate in response to superantigen exposure [67]. Two case reports have further suggested a role for *Staphylococcus* in CTCL [68]. Swabs from erythrodermic skin were positive for *Staphylococcus*, and decolonization with antibiotics and petrolatum-containing acetic acid was attempted. One patient was successfully decolonized and was doing well at the time of publication; the other was not successfully decolonized and ultimately passed away after developing disseminated intravascular coagulation. MRSA was found in his peritoneal fluid. Later studies further correlated the presence of *S. aureus* superantigens with the incidence of erythrodermic CTCL [69] and also demonstrated and increased the rate of colonization in patients with CTCL compared to healthy individuals [70]. Further work has demonstrated that *Staphylococcus* exotoxins may lead malignant T-cells to produce interleukin-10, a cytokine that can reduce the immune response and has also been associated with progressive CTCL that is resistant to treatment [71]. Another case-control study looked at 310 individuals with CTCL with matched controls and found an elevated risk (odds ratio 3.33) of CTCL with a history of impetigo 1 to 5 years before diagnosis, further suggesting a possible role for bacteria in the development or persistence of the disease [72].

There has been one study investigating the role of decolonization in CTCL [73]. Of 106 patients sampled, 59% and 54% were colonized in the skin and nares, respectively, by *Staphylococcus.* Specifically, *Staphylococcus aureus* colonized 31% of skin- and nare-positive patients. Patients were treated with intranasal mupirocin twice daily for 5 days, then weekly, in addition to oral antibiotics for 4 weeks (dicloxacillin or cefalexin for penicillin-allergic patients). Over half of the treated patients demonstrated a clinical response to antibiotic therapy by having a reduction in BSA of at least 50%, with some patients progressing to a complete response, or 100% reduction in affected BSA. Antibiotics eradicated *S. aureus* colonization in up to 91% of patients treated at 4 to 8 weeks of follow-up. Flaring of CTCL was noted with recolonization. It would be interesting to follow patients for a longer period to see how long the decolonization protocol can improve or maintain remission of their disease, as well as using antibiotic-free methods of decolonization, such as bleach baths. Regardless, this study is important to the field as it has correlated clinical improvement with *Staphylococcus* decolonization in patients with CTCL.

### 2.5. Mohs Surgery

The risk of infection with Mohs surgery is considered low but varies with patient and environmental risk factors. Some cases deemed high risk will be treated with prophylactic antibiotics [74]. The World Health Organization recommends preoperative decolonization with twice daily intranasal mupirocin, with or without chlorhexidine wash, for prevention of surgical site infections in individuals who have had a positive *Staphylococcus* nasal swab [75]. Nasal colonization is the most important risk factor for surgical site infections in patients [76]. An RCT of 738 Mohs surgery cases randomized *S. aureus* carriers to decolonization treatment (consisting of intranasal mupirocin ointment twice daily and chlorhexidine wash daily for 5 days) or to no pretreatment, with surgical site infection noted as a clinical outcome [76]. 4% of colonized individuals who underwent decolonization developed a surgical site infection compared to 11% of carriers who were not subjected to decolonization. The frequency of surgical site infection in decolonized patients was similar to the 3% rate observed in noncolonized patients. The authors also performed a cost analysis and determined that it would be cheaper to give all patients prophylactic systemic antibiotics instead of swabbing and decolonizing patients, but caution that there are risks on an individual and on a population level by exposing individuals to unnecessary systemic antibiotics. Cited costs include taking time off for screening, swab materials, laboratory costs, and decolonization treatments. Around the same time, a RCT was published comparing the role of prophylactic antibiotics and decolonization in *S. aureus* 693 carriers undergoing Mohs surgery [77]. Prophylactic antibiotics consisted of 2 doses of oral cefalexin. 9% of patients receiving prophylaxis developed a surgical site infection, whereas no patients in the decolonization group developed an infection. Although this study was not blinded, it supports using decolonization procedures for identified carriers if the associated costs are within reason.

Despite *Staphylococcus* preferentially colonizing the nares and nasal passages, there is concern that nasal swabs may underreport true carriers, as organisms have been found elsewhere on nasal swab-negative patients. A recent RCT recruited 1350 nasal swab-negative patients and randomized to decolonization with twice daily intranasal mupirocin and daily chlorhexidine wash for 5 days vs no treatment [78]. At 1 week, 2% of patients who underwent decolonization had a surgical site infection compared to 4% of controls. Similarly, this study could not be blinded. Taken together, there appears to be a role in decolonization regardless of the results of a nasal swab with the overall trend towards reducing surgical site infection in Mohs patients.

## 3. Antimicrobial Resistance

Not surprisingly, there is growing bacterial resistance to mupirocin. One hospital saw their prevalence of mupirocin resistance in MRSA isolates increase from 2.7% to 65% after they started a mupirocin decolonization protocol [79]. It is important to note that this study observed a rapid rise of mupirocin resistance during a MRSA epidemic in a teaching hospital when mupiroin ointment was used intranasally three times per day every day during hospital admission. High rates of resistance have been associated with long-term use of mupirocin [80]. Other studies have identified hospitals and community homes with high rates of resistance [81], and resistance is associated with failure of decolonization [82]. Similarly, the prevalence of chlorhexidine resistance is increasing, with reported resistance rates of up to 19.3% in the intensive care unit setting [83]. Most studies examining the development of resistance have focused on hospital wards and intensive care units. There is a call for antiseptic stewardship, wherein one would restrict use of antiseptics for nonevidence-based applications [84]. In the era of antibiotic resistance, it remains to be seen whether the morbidity and financial benefits of decolonization can outweigh the risks associated with resistance to these agents. As there has not been reported resistance to bleach baths, it is possible that this could substitute for chlorhexidine washes in future decolonization protocols.

## 4. Future Studies and Conclusions

The Infectious Disease Society of America has published recommended guidelines for recurrent MRSA infections, which include 5 to 10 days of intranasal mupirocin, with body decolonization with daily chlorhexidine washes for 5 to 14 days or 15-minute dilute bleach baths twice per week for 3 months [85]. These recommendations are based on evidence relating to SSTI, as well as endocarditis and central nervous system infections. This regimen is common in the studies discussed and appears to have the ability to successfully decolonize patients. In the context of antimicrobial resistance, there is a growing need for successful decolonization strategies that can maintain long-term eradication.

*S. aureus* is a particularly troublesome organism in dermatology. It has been implicated in the pathogenesis and persistence of multiple skin conditions. Decolonizing affected individuals may have a positive effect on their disease, in addition to preventing recurrent infections. Tailored decolonization protocols may be best depending on the patient's condition.

## References

[1] Mermel L. A., Cartony J. M., Covington P., Maxey G., Morse D. (2011). Methicillin-resistant *Staphylococcus aureus* colonization at different body sites: a prospective, quantitative analysis. *Journal of Clinical Microbiology*.

[2] Kaspar U., Kriegeskorte A., Schubert T. (2016). The culturome of the human nose habitats reveals individual bacterial fingerprint patterns. *Environmental Microbiology*.

[3] von Eiff C., Becker K., Machka K., Stammer H., Peters G. (2001). Nasal carriage as a source of *Staphylococcus aureus* bacteremia. Study Group. *New England Journal of Medicine*.

[4] Klevens R. M., Morrison M. A., Nadle J. (2007). Invasive methicillin-resistant *Staphylococcus aureus* infections in the United States. *JAMA*.

[5] Lindsay J. A., Holden M. T. (2004). *Staphylococcus aureus*: superbug, super genome?. *Trends in Microbiology*.

[6] Miller R. R., Walker A. S., Godwin H. (2014). Dynamics of acquisition and loss of carriage of *Staphylococcus aureus* strains in the community: the effect of clonal complex. *Journal of Infection*.

[7] Barrett F. F., McGehee R. F., Finland M. (1968). Methicillin-resistant *Staphylococcus aureus* at Boston city hospital. *New England Journal of Medicine*.

[8] Tidwell J., Kirk L., Luttrell T., Pike C. A. (2016). CA-MRSA decolonization strategies: do they reduce recurrence rate?. *Journal of Wound, Ostomy and Continence Nursing*.

[9] McKinnell J. A., Huang S. S., Eells S. J., Cui E., Miller L. G. (2013). Quantifying the impact of extranasal testing of body sites for methicillin-resistant *Staphylococcus aureus* colonization at the time of hospital or intensive care unit admission. *Infection Control and Hospital Epidemiology*.

[10] Grothe C., Taminato M., Belasco A., Sesso R., Barbosa D. (2016). Prophylactic treatment of chronic renal disease in patients undergoing peritoneal dialysis and colonized by *Staphylococcus aureus*: a systematic review and meta-analysis. *BMC Nephrology*.

[11] Levy P. Y., Ollivier M., Drancourt M., Raoult D., Argenson J. N. (2013). Relation between nasal carriage of *Staphylococcus aureus* and surgical site infection in orthopedic surgery: the role of nasal contamination. A systematic literature review and meta-analysis. *Orthopaedics and Traumatology: Surgery and Research*.

[12] Weiser M. C., Moucha C. S. (2015). The current state of screening and decolonization for the prevention of *Staphylococcus aureus* surgical site infection after total hip and knee arthroplasty. *Journal of Bone and Joint Surgery-American Volume*.

[13] Tom T. S., Kruse M. W., Reichman R. T. (2009). Update: methicillin-resistant *Staphylococcus aureus* screening and decolonization in cardiac surgery. *Annals of Thoracic Surgery*.

[14] Schweizer M., Perencevich E., McDanel J. (2013). Effectiveness of a bundled intervention of decolonization and prophylaxis to decrease gram positive surgical site infections after cardiac or orthopedic surgery: systematic review and meta-analysis. *BMJ*.

[15] O’Reilly E. B., Johnson M. D., Rohrich R. J. (2014). Comprehensive review of methicillin-resistant *Staphylococcus aureus*: screening and preventive recommendations for plastic surgeons and other surgical health care providers. *Plastic and Reconstructive Surgery*.

[16] Kavanagh K. T., Calderon L. E., Saman D. M., Abusalem S. K. (2014). The use of surveillance and preventative measures for methicillin-resistant staphylococcus aureus infections in surgical patients. *Antimicrobial Resistance and Infection Control*.

[17] Humphreys H., Becker K., Dohmen P. M. (2016). *Staphylococcus aureus* and surgical site infections: benefits of screening and decolonization before surgery. *Journal of Hospital Infection*.

[18] Henriksen L., Simonsen J., Haerskjold A. (2015). Incidence rates of atopic dermatitis, asthma, and allergic rhinoconjunctivitis in Danish and Swedish children. *Journal of Allergy and Clinical Immunology*.

[19] Carroll C. L., Balkrishnan R., Feldman S. R., Fleischer A. B., Manuel J. C. (2005). The burden of atopic dermatitis: impact on the patient, family, and society. *Pediatric Dermatology*.

[20] Cai S. C., Chen H., Koh W. P. (2012). Filaggrin mutations are associated with recurrent skin infection in Singaporean Chinese patients with atopic dermatitis. *British Journal of Dermatology*.

[21] Zollner T. M., Wichelhaus T. A., Hartung A. (2000). Colonization with superantigen-producing *Staphylococcus aureus* is associated with increased severity of atopic dermatitis. *Clinical Experimental Allergy*.

[22] Abeck D., Mempel M. (1998). *Staphylococcus aureus* colonization in atopic dermatitis and its therapeutic implications. *British Journal of Dermatology*.

[23] Gong J. Q., Lin L., Lin T. (2006). Skin colonization by *Staphylococcus aureus* in patients with eczema and atopic dermatitis and relevant combined topical therapy: a double-blind multicentre randomized controlled trial. *British Journal of Dermatology*.

[24] Petry V., Bessa G. R., Poziomczyck C. S. (2012). Bacterial skin colonization and infections in patients with atopic dermatitis. *Anais Brasileiros de Dermatologia*.

[25] Blazewicz I., Jaśkiewicz M., Bauer M. (2017). Decolonization of *Staphylococcus aureus* in patients with atopic dermatitis: a reason for increasing resistance to antibiotics?. *Advances in Dermatology and Allergology*.

[26] Totté J. E., van der Feltz W. T., Hennekam M., van Belkum A., van Zuuren E. J., Pasmans S. G. (2016). Prevalence and odds of *Staphylococcus aureus* carriage in atopic dermatitis: a systematic review and meta-analysis. *British Journal of Dermatology*.

[27] Meylan P., Lang C., Mermoud S. (2017). Skin colonization by *Staphylococcus aureus* precedes the clinical diagnosis of atopic dermatitis in infancy. *Journal of Investigative Dermatology*.

[28] Arikawa J., Ishibashi M., Kawashima M., Takagi Y., Ichikawa Y., Imokawa G. (2002). Decreased levels of sphingosine, a natural antimicrobial agent, may be associated with vulnerability of the stratum corneum from patients with atopic dermatitis to colonization by *Staphylococcus aureus*. *Journal of Investigative Dermatology*.

[29] Li S., Villarreal M., Stewart S. (2017). Altered composition of epidermal lipids correlates with *Staphylococcus aureus* colonization status in atopic dermatitis. *British Journal of Dermatology*.

[30] Komatsu N., Saijoh K., Kuk C. (2007). Human tissue kallikrein expression in the stratum corneum and serum of atopic dermatitis patients. *Experimental Dermatology*.

[31] Strange P., Skov L., Lisby S., Nielsen P. L., Baadsgaard O. (1996). Staphylococcal enterotoxin B applied on intact normal and intact atopic skin induces dermatitis. *Archives of Dermatology*.

[32] Ong P. Y., Ohtake T., Brandt C. (2002). Endogenous antimicrobial peptides and skin infections in atopic dermatitis. *New England Journal of Medicine*.

[33] Brown S. J., McLean W. H. (2012). One remarkable molecule: filaggrin. *Journal of Investigative Dermatology*.

[34] Clausen M. L., Edslev S. M., Andersen P. S., Clemmensen K., Krogfelt K. A., Agner T. (2017). *Staphylococcus aureus* colonization in atopic eczema and its association with filaggrin gene mutations. *British Journal of Dermatology*.

[35] Clausen M. L., Agner T., Lilje B., Edslev S. M., Johannesen T. B., Andersen P. S. (2018). Association of disease severity with skin microbiome and filaggrin gene mutations in adult atopic dermatitis. *JAMA Dermatology*.

[36] Williams M. R., Gallo R. L. (2015). The role of the skin microbiome in atopic dermatitis. *Current Allergy and Asthma Reports*.

[37] Barnes T. M., Greive K. A. (2013). Use of bleach baths for the treatment of infected atopic eczema. *Australasian Journal of Dermatology*.

[38] Rutala W. A., Cole E. C., Thomann C. A., Weber D. J. (1998). Stability and bactericidal activity of chlorine solutions. *Infection Control and Hospital Epidemiology*.

[39] Krakowski A. C., Eichenfield L. F., Dohil M. A. (2008). Management of atopic dermatitis in the pediatric population. *Pediatrics*.

[40] Bath-Hextall F. J., Birnie A. J., Ravenscroft J. C., Williams H. C. (2010). Interventions to reduce *Staphylococcus aureus* in the management of atopic eczema: an updated cochrane review. *British Journal of Dermatology*.

[41] Huang J. T., Abrams M., Tlougan B., Rademaker A., Paller A. S. (2009). Treatment of *Staphylococcus aureus* colonization in atopic dermatitis decreases disease severity. *Pediatrics*.

[42] Fritz S. A., Camins B. C., Eisenstein K. A. (2011). Effectiveness of measures to eradicate *Staphylococcus aureus* carriage in patients with community-associated skin and soft-tissue infections: a randomized trial. *Infection Control and Hospital Epidemiology*.

[43] Hersh A. L., Chambers H. F., Maselli J. H., Gonzales R. (2008). National trends in ambulatory visits and antibiotic prescribing for skin and soft-tissue infections. *Archives of Internal Medicine*.

[44] Witt W. P., Weiss A. J., Elixhauser A. (2006). *Overview of Hospital Stays for Children in the United States, 2012: Statistical Brief #187*.

[45] Mascitti K. B., Gerber J. S., Zaoutis T. E., Barton T. D., Lautenbach E. (2010). Preferred treatment and prevention strategies for recurrent community-associated methicillin-resistant *Staphylococcus aureus* skin and soft-tissue infections: a survey of adult and pediatric providers. *American Journal of Infection Control*.

[46] Miller L. G., Eells S. J., Taylor A. R. (2012). *Staphylococcus aureus* colonization among household contacts of patients with skin infections: risk factors, strain discordance, and complex ecology. *Clinical Infectious Diseases*.

[47] Rodriguez M., Hogan P. G., Krauss M., Warren D. K., Fritz S. A. (2013). Measurement and impact of *Staphylococcus aureus* colonization pressure in households. *Journal of Pediatric Infectious Diseases Society*.

[48] Fritz S. A., Hogan P. G., Hayek G. (2012). Household versus individual approaches to eradication of community-associated *Staphylococcus aureus* in children: a randomized trial. *Clinical Infectious Diseases*.

[49] Tzermpos F., Kanni T., Tzanetakou V. (2013). An algorithm for the management of *Staphylococcus aureus* carriage within patients with recurrent staphylococcal skin infections. *Journal of Infection and Chemotherapy*.

[50] Aiello A. E., Lowy F. D., Wright L. N., Larson E. L. (2006). Meticillin-resistant *Staphylococcus aureus* among US prisoners and military personnel: review and recommendations for future studies. *Lancet Infectious Diseases*.

[51] Morrison S. M., Blaesing C. R., Millar E. V. (2013). Evaluation of methicillin-resistant *Staphylococcus aureus* skin and soft-tissue infection prevention strategies at a military training center. *Infection Control and Hospital Epidemiology*.

[52] Ellis M. W., Schlett C. D., Millar E. V. (2014). Hygiene strategies to prevent methicillin-resistant *Staphylococcus aureus* skin and soft tissue infections: a cluster-randomized controlled trial among high-risk military trainees. *Clinical Infectious Diseases*.

[53] Millar E. V., Chen W. J., Schlett C. D. (2015). Frequent use of chlorhexidine-based body wash associated with a reduction in methicillin-resistant *Staphylococcus aureus* nasal colonization among military trainees. *Antimicrobial Agents and Chemotherapy*.

[54] Schmitz G. R. (2011). How do you treat an abscess in the era of increased community-associated methicillin-resistant *Staphylococcus aureus* (MRSA)?. *Journal of Emergency Medicine*.

[55] Duong M., Markwell S., Peter J., Barenkamp S. (2010). Randomized, controlled trial of antibiotics in the management of community-acquired skin abscesses in the pediatric patient. *Annals of Emergency Medicine*.

[56] Schmitz G. R., Bruner D., Pitotti R. (2010). Randomized controlled trial of trimethoprim-sulfamethoxazole for uncomplicated skin abscesses in patients at risk for community-associated methicillin-resistant *Staphylococcus aureus* infection. *Annals of Emergency Medicine*.

[57] Hogan P. G., Rodriguez M., Spenner A. M. (2018). Impact of systemic antibiotics on *Staphylococcus aureus* colonization and recurrent skin infection. *Clinical Infectious Diseases*.

[58] Mortz C. G., Bindslev-Jensen C., Andersen K. E. (2014). Hand eczema in the odense adolescence cohort study on atopic diseases and dermatitis (TOACS): prevalence, incidence and risk factors from adolescence to adulthood. *British Journal of Dermatology*.

[59] von Kobyletzki L. B., Beckman L., Smeeth L. (2017). Association between childhood allergic diseases, educational attainment and occupational status in later life: systematic review protocol. *BMJ Open*.

[60] Haslund P., Bangsgaard N., Jarløv J. O., Skov L., Skov R., Agner T. (2009). *Staphylococcus aureus* and hand eczema severity. *British Journal of Dermatology*.

[61] Mernelius S., Carlsson E., Henricson J. (2016). *Staphylococcus aureus* colonization related to severity of hand eczema. *European Journal of Clinical Microbiology and Infectious Diseases*.

[62] Lundov M. D., Johansen J. D., Zachariae C., Moesby L. (2012). Creams used by hand eczema patients are often contaminated with *Staphylococcus aureus*. *Acta Dermato Venereologica*.

[63] Brannan D. K., Dille J. C. (1990). Type of closure prevents microbial contamination of cosmetics during consumer use. *Applied and Environmental Microbiology*.

[64] Heald P., Edelson R. (1991). Immunology of cutaneous T-cell lymphoma. *Journal of National Cancer Institute*.

[65] Bahler D. W., Berry G., Oksenberg J., Warnke R. A., Levy R. (1992). Diversity of T-cell antigen receptor variable genes used by mycosis fungoides cells. *American Journal of Pathology*.

[66] Janeway C. A. (1991). Selective elements for the V beta region of the T cell receptor: Mls and the bacterial toxic mitogens. *Advances in Immunology*.

[67] Tokura Y., Heald P. W., Yan S. L., Edelson R. L. (1992). Stimulation of cutaneous T-cell lymphoma cells with superantigenic staphylococcal toxins. *Journal of Investigative Dermatology*.

[68] Tokura Y., Yagi H., Ohshima A. (1995). Cutaneous colonization with staphylococci influences the disease activity of Sézary syndrome: a potential role for bacterial superantigens. *British Journal of Dermatology*.

[69] Jackow C. M., Cather J. C., Hearne V., Asano A. T., Musser J. M., Duvic M. (1997). Association of erythrodermic cutaneous T-cell lymphoma, superantigen-positive *Staphylococcus aureus*, and oligoclonal T-cell receptor V beta gene expansion. *Blood*.

[70] Nguyen V., Huggins R. H., Lertsburapa T. (2008). Cutaneous T-cell lymphoma and *Staphylococcus aureus* colonization. *Journal of American Academy of Dermatology*.

[71] Krejsgaard T., Willerslev-Olsen A., Lindahl L. M. (2014). Staphylococcal enterotoxins stimulate lymphoma-associated immune dysregulation. *Blood*.

[72] Boursi B., Haynes K., Mamtani R., Yang Y. X. (2016). An association between newly diagnosed cutaneous T cell lymphoma and prior impetigo: a nested case-control study. *Archives of Dermatological Research*.

[73] Talpur R., Bassett R., Duvic M. (2008). Prevalence and treatment of *Staphylococcus aureus* colonization in patients with mycosis fungoides and Sezary syndrome. *British Journal of Dermatology*.

[74] Maragh S. L., Brown M. D. (2008). Prospective evaluation of surgical site infection rate among patients with Mohs micrographic surgery without the use of prophylactic antibiotics. *Journal of American Academy of Dermatology*.

[75] WHO (2016). Decolonization with mupirocin ointment with or without chlorhexidine gluconate body wash for the prevention of *Staphylococcus aureus* infection in nasal carriers undergoing surgery. *Global Guidelines for the Prevention of Surgical Site Infection*.

[76] Tai Y. J., Borchard K. L., Gunson T. H., Smith H. R., Vinciullo C. (2013). Nasal carriage of *Staphylococcus aureus* in patients undergoing Mohs micrographic surgery is an important risk factor for postoperative surgical site infection: a prospective randomised study. *Australasian Journal of Dermatology*.

[77] Cherian P., Gunson T., Borchard K., Tai Y., Smith H., Vinciullo C. (2013). Oral antibiotics versus topical decolonization to prevent surgical site infection after Mohs micrographic surgery--a randomized, controlled trial. *Dermatologic Surgery*.

[78] Smith H., Borchard K., Cherian P., Tai Y., Vinciullo C. (2018). Randomized controlled trial of preoperative topical decolonization to reduce surgical site infection for *Staphylococcus aureus* nasal swab-negative Mohs micrographic surgery patients. *Dermatologic Surgery*.

[79] Miller M. A., Dascal A., Portnoy J., Mendelson J. (1996). Development of mupirocin resistance among methicillin-resistant *Staphylococcus aureus* after widespread use of nasal mupirocin ointment. *Infection Control and Hospital Epidemiology*.

[80] Wise R., Johnson J. (1991). Mupirocin resistance. *Lancet*.

[81] Madden G. R., Sifri C. D. (2018). Antimicrobial resistance to agents used for *Staphylococcus aureus* decolonization: is there a reason for concern?. *Curr Infect Dis Rep*.

[82] Simor A. E., Phillips E., McGeer A. (2007). Randomized controlled trial of chlorhexidine gluconate for washing, intranasal mupirocin, and rifampin and doxycycline versus no treatment for the eradication of methicillin-resistant *Staphylococcus aureus* colonization. *Clinical Infectious Diseases*.

[83] Cho O. H., Park K.-H., Song J. Y. (2018). Prevalence and microbiological characteristics of qacA/B-positive methicillin-resistant *Staphylococcus aureus* isolates in a surgical intensive care unit. *Microbial Drug Resistance*.

[84] Kampf G. (2016). Acquired resistance to chlorhexidine-is it time to establish an antiseptic stewardship’ initiative?. *Journal of Hospital Infection*.

[85] Liu C., Bayer A., Cosgrove S. E. (2011). Clinical practice guidelines by the infectious diseases society of America for the treatment of methicillin-resistant *Staphylococcus aureus* infections in adults and children: executive summary. *Clinical Infectious Diseases*.

